# 2-Amino-1*H*-benzoimidazol-3-ium 4,4,4-trifluoro-1,3-dioxo-1-phenyl­butan-2-ide

**DOI:** 10.1107/S1600536808037483

**Published:** 2008-11-26

**Authors:** Gong-Chun Li, Feng-Ling Yang, Chang-Sheng Yao

**Affiliations:** a College of Chemistry and Chemical Engineering, Xuchang University, Xuchang, Henan Province 461000, People’s Republic of China; b School of Chemistry and Chemical Engineering, and Key Laboratory of Biotechnology for Medicinal Plants, Xuzhou Normal University, Xuzhou 221116, People’s Republic of China

## Abstract

In the title compound, C_7_H_8_N_3_^+^·C_10_H_6_F_3_O_2_^−^, 1*H*-benzoimidazol-2-amine system adopts a planar conformation with an r.m.s. deviation of 0.0174 Å. The cation and anion in the asymmetric unit are linked by N—H⋯O hydrogen bonds. There are also additional inter­molecular N—H⋯O hydrogen bonds and π–π stacking inter­actions between the phenyl rings of neighbouring anions with centroid–centroid distances of 4.0976 (13) Å.

## Related literature

For details of the bioactivity of organofluorine compounds, see: Hermann *et al.* (2003 ▶); Ulrich (2004 ▶).
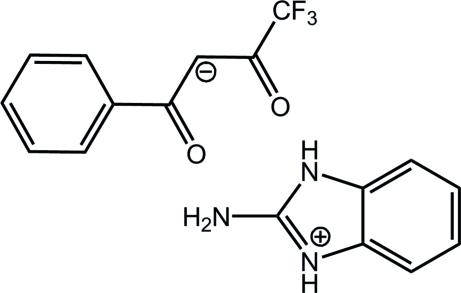


## Experimental

### 

#### Crystal data

C_7_H_8_N_3_^+^·C_10_H_6_F_3_O_2_^−^
*M* *_r_* = 349.31
Orthorhombic, 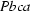

*a* = 16.755 (3) Å
*b* = 10.552 (2) Å
*c* = 18.497 (4) Å
*V* = 3270.0 (11) Å^3^
*Z* = 8
Mo *K*α radiation
μ = 0.12 mm^−1^
*T* = 113 (2) K
0.12 × 0.10 × 0.06 mm

#### Data collection

Rigaku Saturn CCD diffractometer
Absorption correction: multi-scan (*CrystalClear*; Rigaku/MSC, 2002 ▶) *T*_min_ = 0.986, *T*_max_ = 0.993
31132 measured reflections
2881 independent reflections
2609 reflections with *I* > 2σ(*I*)
*R*_int_ = 0.050

#### Refinement

*R*[*F* ^2^ > 2σ(*F* ^2^)] = 0.039
*wR*(*F* ^2^) = 0.107
*S* = 1.06
2881 reflections
243 parameters
5 restraints
H atoms treated by a mixture of independent and constrained refinement
Δρ_max_ = 0.21 e Å^−3^
Δρ_min_ = −0.20 e Å^−3^

### 

Data collection: *CrystalClear* (Rigaku/MSC, 2002 ▶); cell refinement: *CrystalClear*; data reduction: *CrystalClear*; program(s) used to solve structure: *SHELXS97* (Sheldrick, 2008 ▶); program(s) used to refine structure: *SHELXL97* (Sheldrick, 2008 ▶); molecular graphics: *SHELXTL* (Sheldrick, 2008 ▶); software used to prepare material for publication: *SHELXTL*.

## Supplementary Material

Crystal structure: contains datablocks I, global. DOI: 10.1107/S1600536808037483/sj2549sup1.cif

Structure factors: contains datablocks I. DOI: 10.1107/S1600536808037483/sj2549Isup2.hkl

Additional supplementary materials:  crystallographic information; 3D view; checkCIF report

## Figures and Tables

**Table 1:** Hydrogen-bond geometry (Å, °)

*D*—H⋯*A*	*D*—H	H⋯*A*	*D*⋯*A*	*D*—H⋯*A*
N1—H1*A*⋯O2^i^	0.877 (9)	2.004 (11)	2.7803 (18)	146.8 (16)
N3—H3*A*⋯O1^i^	0.895 (9)	1.949 (13)	2.7651 (17)	150.9 (19)
N3—H3*A*⋯O2^i^	0.895 (9)	2.363 (18)	2.9912 (18)	127.3 (17)
N1—H1*B*⋯O1	0.879 (9)	2.030 (10)	2.8662 (18)	158.4 (16)
N2—H2*A*⋯O2	0.905 (10)	1.862 (11)	2.7360 (18)	161.8 (19)

Symmetry code: (i) 
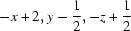
.

**Fig. 1.**
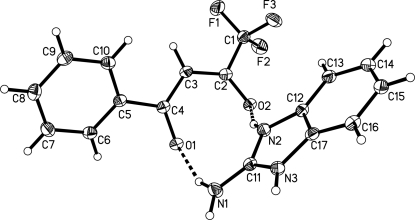
The asymmetric unit of the title compound, showing 30% probability displacement ellipsoids and the atom-numbering scheme. Hydrogen bonds are drawn as dashed lines.

**Fig. 2.**
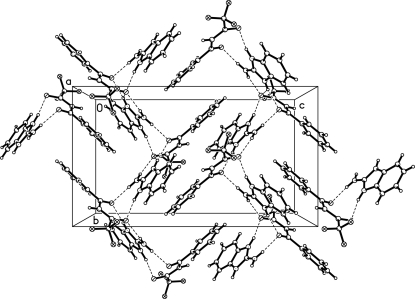
The packing diagram of the title compound. Intermolecular hydrogen bonds are shown as dashed lines.

## supplementary crystallographic information

### Comment

Compounds that contain fluorine exhibit particular bioactivity, for example, flumioxazin is a widely used herbicide (Hermann *et al.*, 2003; Ulrich,2004). This led us to pay much attention to the synthesis and structure of similar fluoro-compounds. During the synthesis of trifluoromethylated heterocyclic compounds, an intermediate, the title compound,(I), Fig.1, was isolated and we report its crystal structure here.

The 1*H*-benzo[*d*]imizol-2-amine cation adopts a planar conformation with an rms deviation of 0.0174 for the fitted atoms. The phenyl ring is almost perpendicular to the fused heterocyclic rings, with a dihedral angle of 81.84 (4)° between them. The cation and anion in the asymmetric unit are linked by N1—H1B···O1 and N2—H2A···O2 hydrogen bonds.

The crystal packing is further stabilized by additional intermolecular N—H···O hydrogen bonds (Table 1, Fig. 2) and intermolecular π···π stacking interactions between the C5-C10 phenyl rings (symmetry code: 2-x, -y, -z) of neighbouring anions with centroid-to-centroid distances, plane-plane distances and displacement distances of 4.0976 (13), 3.786 and 1.565Å respectively.

### Experimental

The title compound was synthesized by the reaction of 4,4,4-trifluoro-1- phenylbutane-1,3-dione (1 mmol) and 1*H*-benzo[*d*]imidazol-2-amine (1 mmol) in refluxing ethanol (20 mL) for a certain time (monitored by TLC). Cooling, the reaction mixture slowly to room temperature, gave single crystals suitable for X-ray diffraction.

### Refinement

Hydrogen atoms bound to nitrogen atoms were located in a difference Fourier map and were refined with d(N—H) restrained to 0.90 (1) Å and d(H1A···H1B) restrained to 1.50 (1) Å. Other H atoms were placed in calculated positions, d(C—H) = 0.93 Å, and included in the final cycles of refinement using a riding model, with *U*_iso_(H) = 1.2*U*_eq_(C).

### Crystal data

**Table:**

C_7_H_8_N_3_^+^·C_10_H_6_F_3_O_2_^–^	*F*_000_ = 1440
*M**_r_* = 349.31	*D*_x_ = 1.419 Mg m^−^^3^
Orthorhombic, *P**b**c**a*	Mo *K*α radiation λ = 0.71073 Å
Hall symbol: -P 2ac 2ab	Cell parameters from 6974 reflections
*a* = 16.755 (3) Å	θ = 2.2–27.9º
*b* = 10.552 (2) Å	µ = 0.12 mm^−^^1^
*c* = 18.497 (4) Å	*T* = 113 (2) K
*V* = 3270.0 (11) Å^3^	Block, yellow
*Z* = 8	0.12 × 0.10 × 0.06 mm

### Data collection

**Table:**

Rigaku Saturn CCD diffractometer	2881 independent reflections
Radiation source: rotating anode	2609 reflections with *I* > 2σ(*I*)
Monochromator: confocal	*R*_int_ = 0.050
*T* = 113(2) K	θ_max_ = 25.0º
ω scans	θ_min_ = 2.2º
Absorption correction: multi-scan(CrystalClear; Rigaku/MSC, 2002)	*h* = −19→19
*T*_min_ = 0.986, *T*_max_ = 0.993	*k* = −12→12
31132 measured reflections	*l* = −22→21

### Refinement

**Table:**

Refinement on *F*^2^	Secondary atom site location: difference Fourier map
Least-squares matrix: full	Hydrogen site location: inferred from neighbouring sites
*R*[*F*^2^ > 2σ(*F*^2^)] = 0.039	H atoms treated by a mixture of independent and constrained refinement
*wR*(*F*^2^) = 0.107	*w* = 1/[σ^2^(*F*_o_^2^) + (0.0613*P*)^2^ + 0.895*P*] where *P* = (*F*_o_^2^ + 2*F*_c_^2^)/3
*S* = 1.06	(Δ/σ)_max_ = 0.001
2881 reflections	Δρ_max_ = 0.21 e Å^−^^3^
243 parameters	Δρ_min_ = −0.20 e Å^−^^3^
5 restraints	Extinction correction: SHELXL97 (Sheldrick, 2008), Fc^*^=kFc[1+0.001xFc^2^λ^3^/sin(2θ)]^-1/4^
Primary atom site location: structure-invariant direct methods	Extinction coefficient: 0.0073 (10)

### Special details

**Table:**

Geometry. All esds (except the esd in the dihedral angle between two l.s. planes) are estimated using the full covariance matrix. The cell esds are taken into account individually in the estimation of esds in distances, angles and torsion angles; correlations between esds in cell parameters are only used when they are defined by crystal symmetry. An approximate (isotropic) treatment of cell esds is used for estimating esds involving l.s. planes.
Refinement. Refinement of F^2^ against ALL reflections. The weighted R-factor wR and goodness of fit S are based on F^2^, conventional R-factors R are based on F, with F set to zero for negative F^2^. The threshold expression of F^2^ > 2sigma(F^2^) is used only for calculating R-factors(gt) etc. and is not relevant to the choice of reflections for refinement. R-factors based on F^2^ are statistically about twice as large as those based on F, and R- factors based on ALL data will be even larger.

### Fractional atomic coordinates and isotropic or equivalent isotropic displacement parameters (Å^2^)

**Table:**

	*x*	*y*	*z*	*U*_iso_*/*U*_eq_	
F1	0.74257 (7)	0.54639 (12)	0.05657 (5)	0.0552 (3)	
F2	0.77552 (6)	0.66729 (9)	0.14532 (6)	0.0473 (3)	
F3	0.71669 (6)	0.49082 (11)	0.16580 (6)	0.0467 (3)	
O1	1.01117 (6)	0.37243 (10)	0.11875 (6)	0.0301 (3)	
O2	0.88568 (6)	0.49842 (10)	0.19269 (6)	0.0303 (3)	
N1	1.03832 (8)	0.19158 (15)	0.23172 (8)	0.0379 (4)	
N2	0.90662 (8)	0.26462 (13)	0.25377 (7)	0.0298 (3)	
N3	0.94668 (8)	0.09546 (13)	0.31266 (7)	0.0295 (3)	
C1	0.77211 (10)	0.54628 (16)	0.12397 (9)	0.0335 (4)	
C2	0.85307 (9)	0.48052 (14)	0.13136 (8)	0.0277 (3)	
C3	0.88043 (9)	0.40780 (15)	0.07420 (8)	0.0290 (4)	
H3	0.8467	0.3955	0.0349	0.035*	
C4	0.95774 (9)	0.35055 (14)	0.07232 (8)	0.0264 (3)	
C5	0.97828 (9)	0.26266 (14)	0.01141 (8)	0.0263 (3)	
C6	1.05759 (10)	0.25313 (16)	−0.01066 (9)	0.0324 (4)	
H6	1.0962	0.3030	0.0116	0.039*	
C7	1.07968 (10)	0.17084 (17)	−0.06508 (10)	0.0378 (4)	
H7	1.1327	0.1665	−0.0797	0.045*	
C8	1.02270 (11)	0.09443 (16)	−0.09797 (9)	0.0368 (4)	
H8	1.0376	0.0379	−0.1341	0.044*	
C9	0.94389 (10)	0.10278 (15)	−0.07679 (9)	0.0346 (4)	
H9	0.9057	0.0518	−0.0989	0.042*	
C10	0.92111 (10)	0.18696 (15)	−0.02263 (8)	0.0308 (4)	
H10	0.8678	0.1928	−0.0091	0.037*	
C11	0.96806 (9)	0.18451 (15)	0.26443 (8)	0.0296 (4)	
C12	0.84329 (9)	0.22790 (15)	0.29854 (8)	0.0275 (4)	
C13	0.76879 (9)	0.28105 (17)	0.31021 (8)	0.0321 (4)	
H13	0.7518	0.3527	0.2853	0.038*	
C14	0.72078 (10)	0.22158 (17)	0.36124 (9)	0.0349 (4)	
H14	0.6705	0.2548	0.3710	0.042*	
C15	0.74579 (10)	0.11347 (17)	0.39833 (9)	0.0339 (4)	
H15	0.7118	0.0763	0.4319	0.041*	
C16	0.82038 (10)	0.06027 (15)	0.38614 (8)	0.0309 (4)	
H16	0.8371	−0.0120	0.4106	0.037*	
C17	0.86862 (9)	0.11999 (14)	0.33575 (8)	0.0273 (3)	
H1A	1.0783 (9)	0.1439 (16)	0.2457 (10)	0.046 (5)*	
H1B	1.0438 (10)	0.2461 (14)	0.1961 (8)	0.043 (5)*	
H2A	0.9093 (12)	0.3377 (13)	0.2283 (10)	0.051 (6)*	
H3A	0.9769 (11)	0.0317 (15)	0.3290 (11)	0.051 (6)*	

### Atomic displacement parameters (Å^2^)

**Table:**

	*U*^11^	*U*^22^	*U*^33^	*U*^12^	*U*^13^	*U*^23^
F1	0.0509 (7)	0.0792 (8)	0.0356 (6)	0.0313 (6)	−0.0129 (5)	−0.0053 (6)
F2	0.0462 (6)	0.0331 (6)	0.0628 (7)	0.0093 (4)	0.0013 (5)	−0.0034 (5)
F3	0.0293 (5)	0.0561 (7)	0.0546 (7)	−0.0022 (5)	0.0024 (5)	0.0043 (5)
O1	0.0292 (6)	0.0319 (6)	0.0291 (6)	−0.0017 (5)	−0.0052 (5)	−0.0008 (5)
O2	0.0307 (6)	0.0322 (6)	0.0279 (6)	−0.0019 (5)	−0.0042 (4)	−0.0015 (5)
N1	0.0316 (8)	0.0461 (9)	0.0358 (8)	0.0102 (7)	0.0039 (6)	0.0122 (7)
N2	0.0306 (7)	0.0313 (7)	0.0274 (7)	0.0048 (6)	−0.0006 (5)	0.0055 (6)
N3	0.0298 (7)	0.0304 (7)	0.0284 (7)	0.0056 (6)	−0.0008 (5)	0.0029 (6)
C1	0.0353 (9)	0.0355 (9)	0.0296 (8)	0.0026 (7)	−0.0016 (7)	0.0005 (7)
C2	0.0275 (8)	0.0253 (8)	0.0301 (8)	−0.0030 (6)	−0.0033 (6)	0.0048 (6)
C3	0.0295 (8)	0.0312 (8)	0.0263 (8)	0.0009 (6)	−0.0056 (6)	0.0006 (6)
C4	0.0296 (8)	0.0239 (8)	0.0256 (7)	−0.0038 (6)	−0.0027 (6)	0.0053 (6)
C5	0.0291 (8)	0.0251 (7)	0.0249 (7)	−0.0002 (6)	−0.0030 (6)	0.0044 (6)
C6	0.0285 (8)	0.0337 (9)	0.0350 (9)	−0.0024 (7)	−0.0036 (7)	−0.0011 (7)
C7	0.0309 (9)	0.0418 (10)	0.0407 (9)	0.0010 (7)	0.0045 (7)	−0.0035 (8)
C8	0.0452 (10)	0.0324 (9)	0.0327 (9)	0.0025 (7)	0.0014 (7)	−0.0036 (7)
C9	0.0391 (9)	0.0286 (8)	0.0360 (9)	−0.0057 (7)	−0.0058 (7)	−0.0025 (7)
C10	0.0287 (8)	0.0306 (8)	0.0330 (8)	−0.0030 (6)	−0.0021 (6)	0.0020 (7)
C11	0.0301 (9)	0.0336 (8)	0.0250 (8)	0.0036 (7)	−0.0028 (6)	0.0009 (6)
C12	0.0293 (8)	0.0305 (8)	0.0227 (7)	−0.0001 (6)	−0.0027 (6)	−0.0021 (6)
C13	0.0300 (9)	0.0369 (9)	0.0292 (8)	0.0055 (7)	−0.0047 (6)	−0.0011 (7)
C14	0.0260 (8)	0.0453 (10)	0.0332 (9)	0.0007 (7)	−0.0029 (6)	−0.0045 (8)
C15	0.0300 (8)	0.0421 (10)	0.0298 (8)	−0.0074 (7)	−0.0005 (7)	−0.0015 (7)
C16	0.0348 (9)	0.0300 (8)	0.0279 (8)	−0.0032 (7)	−0.0049 (6)	0.0018 (6)
C17	0.0279 (8)	0.0290 (8)	0.0249 (7)	0.0008 (6)	−0.0056 (6)	−0.0033 (6)

### Geometric parameters (Å, °)

**Table:**

F1—C1	1.3415 (19)	C5—C10	1.397 (2)
F2—C1	1.338 (2)	C6—C7	1.380 (2)
F3—C1	1.3429 (19)	C6—H6	0.9300
O1—C4	1.2618 (18)	C7—C8	1.390 (3)
O2—C2	1.2732 (18)	C7—H7	0.9300
N1—C11	1.326 (2)	C8—C9	1.380 (2)
N1—H1A	0.877 (9)	C8—H8	0.9300
N1—H1B	0.879 (9)	C9—C10	1.392 (2)
N2—C11	1.346 (2)	C9—H9	0.9300
N2—C12	1.401 (2)	C10—H10	0.9300
N2—H2A	0.905 (10)	C12—C13	1.385 (2)
N3—C11	1.344 (2)	C12—C17	1.396 (2)
N3—C17	1.400 (2)	C13—C14	1.390 (2)
N3—H3A	0.895 (9)	C13—H13	0.9300
C1—C2	1.530 (2)	C14—C15	1.395 (2)
C2—C3	1.385 (2)	C14—H14	0.9300
C3—C4	1.430 (2)	C15—C16	1.388 (2)
C3—H3	0.9300	C15—H15	0.9300
C4—C5	1.499 (2)	C16—C17	1.385 (2)
C5—C6	1.394 (2)	C16—H16	0.9300
			
C11—N1—H1A	120.8 (12)	C8—C7—H7	120.0
C11—N1—H1B	118.1 (11)	C9—C8—C7	119.73 (16)
H1A—N1—H1B	121.1 (14)	C9—C8—H8	120.1
C11—N2—C12	108.60 (13)	C7—C8—H8	120.1
C11—N2—H2A	125.0 (13)	C8—C9—C10	120.49 (15)
C12—N2—H2A	125.5 (13)	C8—C9—H9	119.8
C11—N3—C17	108.79 (13)	C10—C9—H9	119.8
C11—N3—H3A	126.8 (14)	C9—C10—C5	120.07 (15)
C17—N3—H3A	124.4 (14)	C9—C10—H10	120.0
F2—C1—F1	106.81 (13)	C5—C10—H10	120.0
F2—C1—F3	105.99 (13)	N1—C11—N3	125.36 (14)
F1—C1—F3	106.30 (13)	N1—C11—N2	125.23 (15)
F2—C1—C2	111.64 (13)	N3—C11—N2	109.40 (14)
F1—C1—C2	114.24 (14)	C13—C12—C17	121.81 (14)
F3—C1—C2	111.35 (13)	C13—C12—N2	131.50 (15)
O2—C2—C3	128.36 (14)	C17—C12—N2	106.66 (13)
O2—C2—C1	113.13 (14)	C12—C13—C14	116.39 (15)
C3—C2—C1	118.47 (14)	C12—C13—H13	121.8
C2—C3—C4	123.55 (14)	C14—C13—H13	121.8
C2—C3—H3	118.2	C13—C14—C15	121.95 (15)
C4—C3—H3	118.2	C13—C14—H14	119.0
O1—C4—C3	123.29 (14)	C15—C14—H14	119.0
O1—C4—C5	117.51 (13)	C16—C15—C14	121.39 (15)
C3—C4—C5	119.18 (13)	C16—C15—H15	119.3
C6—C5—C10	118.71 (14)	C14—C15—H15	119.3
C6—C5—C4	118.92 (13)	C17—C16—C15	116.78 (15)
C10—C5—C4	122.34 (14)	C17—C16—H16	121.6
C7—C6—C5	120.98 (15)	C15—C16—H16	121.6
C7—C6—H6	119.5	C16—C17—C12	121.68 (14)
C5—C6—H6	119.5	C16—C17—N3	131.78 (14)
C6—C7—C8	120.01 (16)	C12—C17—N3	106.52 (13)
C6—C7—H7	120.0		
			
F2—C1—C2—O2	48.38 (18)	C4—C5—C10—C9	−177.14 (14)
F1—C1—C2—O2	169.71 (14)	C17—N3—C11—N1	179.40 (15)
F3—C1—C2—O2	−69.86 (17)	C17—N3—C11—N2	−1.38 (17)
F2—C1—C2—C3	−133.69 (15)	C12—N2—C11—N1	−179.13 (15)
F1—C1—C2—C3	−12.4 (2)	C12—N2—C11—N3	1.64 (18)
F3—C1—C2—C3	108.07 (16)	C11—N2—C12—C13	177.01 (16)
O2—C2—C3—C4	−7.8 (3)	C11—N2—C12—C17	−1.26 (17)
C1—C2—C3—C4	174.64 (14)	C17—C12—C13—C14	0.3 (2)
C2—C3—C4—O1	−8.1 (2)	N2—C12—C13—C14	−177.71 (16)
C2—C3—C4—C5	173.70 (14)	C12—C13—C14—C15	−0.6 (2)
O1—C4—C5—C6	−27.7 (2)	C13—C14—C15—C16	0.3 (2)
C3—C4—C5—C6	150.58 (15)	C14—C15—C16—C17	0.3 (2)
O1—C4—C5—C10	150.25 (14)	C15—C16—C17—C12	−0.5 (2)
C3—C4—C5—C10	−31.4 (2)	C15—C16—C17—N3	177.26 (15)
C10—C5—C6—C7	0.1 (2)	C13—C12—C17—C16	0.2 (2)
C4—C5—C6—C7	178.13 (15)	N2—C12—C17—C16	178.68 (14)
C5—C6—C7—C8	−1.0 (3)	C13—C12—C17—N3	−178.06 (14)
C6—C7—C8—C9	1.1 (3)	N2—C12—C17—N3	0.42 (16)
C7—C8—C9—C10	−0.1 (3)	C11—N3—C17—C16	−177.45 (16)
C8—C9—C10—C5	−0.8 (2)	C11—N3—C17—C12	0.57 (16)
C6—C5—C10—C9	0.9 (2)		

### Hydrogen-bond geometry (Å, °)

**Table:**

*D*—H···*A*	*D*—H	H···*A*	*D*···*A*	*D*—H···*A*
N1—H1A···O2^i^	0.877 (9)	2.004 (11)	2.7803 (18)	146.8 (16)
N3—H3A···O1^i^	0.895 (9)	1.949 (13)	2.7651 (17)	150.9 (19)
N3—H3A···O2^i^	0.895 (9)	2.363 (18)	2.9912 (18)	127.3 (17)
N1—H1B···O1	0.879 (9)	2.030 (10)	2.8662 (18)	158.4 (16)
N2—H2A···O2	0.905 (10)	1.862 (11)	2.7360 (18)	161.8 (19)

Symmetry codes: (i) −*x*+2, *y*−1/2, −*z*+1/2.
